# Factors associated with the improvement of body image dissatisfaction of female patients with overweight and obesity during cognitive behavioral therapy

**DOI:** 10.3389/fpsyt.2022.1025946

**Published:** 2022-10-21

**Authors:** Hiroaki Yokoyama, Takehiro Nozaki, Tomoe Nishihara, Ryoko Sawamoto, Gen Komaki, Nobuyuki Sudo

**Affiliations:** ^1^Department of Psychosomatic Medicine, Graduate School of Medical Sciences, Kyushu University, Fukuoka, Japan; ^2^Graduate School of Nutritional Sciences, Nakamura Gakuen University, Fukuoka, Japan; ^3^Department of Psychosomatic Medicine, National Hospital Organization Fukuoka Higashi Medical Center, Koga, Japan; ^4^Department of Psychosomatic Medicine, Fukuoka Dental College, Fukuoka, Japan; ^5^Faculty of Medical Science, Fukuoka International University of Health and Welfare, Fukuoka, Japan

**Keywords:** weight loss, body image, cognitive behavioral therapy, binge eating, depression, obesity

## Abstract

**Background:**

Cognitive behavioral therapy (CBT) has become one of the most commonly used psychotherapeutic treatments for obesity. It stems from CBT for bulimia nervosa and binge eating disorder, which focuses on amelioration of the eating behavior and body image dissatisfaction (BID), but usually does not focus on weight loss. In contrast, CBT for obesity focuses on weight loss, as well as eating behavior and BID. It is at present unclear whether the improvement of BID during CBT for obesity is associated with improvement of factors other than weight loss.

**Objective:**

The purpose of this study was to determine whether improvement of BID during CBT for obesity was associated with improvement of factors other than weight loss.

**Methods:**

One hundred and sixty-five women (BMI 31.8 ± 5.2 kg/m^2^, age 49.3 ± 10.5 years) with overweight or obesity completed a 7-month CBT-based weight loss intervention. BID, depression, anxiety, binge eating, and perfectionism were assessed at both baseline and the end of the intervention through the use of psychological questionnaires.

**Results:**

Percent total weight loss, baseline BID, baseline binge eating disorder (BED), change in depression (Δdepression), Δstate anxiety, Δtrait anxiety, Δbinge eating, and Δperfectionism were significantly correlated with ΔBID. Multiple regression analysis showed that baseline BID, baseline BED, percent total weight loss, Δbinge eating, and Δdepression were independently associated with ΔBID.

**Conclusion:**

Improvement of binge eating, and improvement of depression, as well as weight loss, were independently associated with amelioration of BID.

**Clinical trial registration:**

[https://center6.umin.ac.jp/cgi-open-bin/ctr_e/ctr_view.cgi?recptno=R000008052], identifier [UMIN000006803] and [https://center6.umin.ac.jp/cgi-open-bin/ctr_e/ctr_view.cgi?recptno=R0000 55850], identifier [UMIN000049041].

## Introduction

The etiology of obesity is multifactorial. It is well-known that both psychological and biological factors play an important role in the development of this disease (1). One of the approaches that focuses on psychological factors is cognitive behavioral therapy (CBT). With the development of CBT for bulimia nervosa (BN) and binge eating disorder (BED) (2), it has become one of the most commonly used psychotherapeutic treatments for obesity.

However, CBT for obesity has different targets than those for BN or BED. CBT, when applied for BN or BED, is based on the hypothesis that patients excessively restrict their diet because of their body image dissatisfaction (BID; negative evaluation of one’s body weight and/or shape) (3), leading them eventually to binge-eat. Therefore, CBT for BN and BED focuses on helping patients establish normal eating patterns while teaching them to identify and challenge negative thoughts on their weight and body image; weight loss is usually not focused on here (4, 5). As a result, it has been shown that CBT for BN and BED improves binge eating and BID, but does not bring about weight loss (6–8). In contrast, CBT for obesity focuses on weight loss as well as disordered eating and BID (2, 9), generally resulting in weight loss, improvement of eating behavior and BID (10–13). In the context of obesity treatment, this creates a contrast between CBT and conventional behavioral treatment, which aims for behavioral change and induces weight loss in the short term, but often fails to promote long-term weight loss maintenance. CBT focuses not only on behavioral change for weight loss, but on cognitive change, such as improvement of BID, to help prevent weight regain (9, 14). As a result, it has been reported that improvement of BID is correlated with both weight loss during weight loss treatment and weight change after the treatment (15, 16). However, this raises questions that need to be clarified: Is the improvement of BID during CBT for obesity due to weight loss *per se*? Are there factors related to the improvement of BID that are independent of weight loss? Previous studies that have examined correlates of BID are either cross-sectional studies (17–22) or longitudinal studies of non-CBT interventions, most of which were bariatric surgery (23–28). To the best of our knowledge, no study has longitudinally examined the relation between improvement of BID through CBT and the change in factors other than weight loss.

We have a CBT-based treatment program that puts emphasis on both weight reduction and cognitive change, particularly on improvement of BID. Identifying factors for which improvement is associated with improvement of BID may contribute to both weight loss and prevention of weight regain after treatment. The purpose of this study was to determine if improvement of factors other than weight loss are associated with BID improvement.

For this study, because of the scarcity of previous longitudinal studies that examined factors through which improvement was related to the improvement of BID, we selected candidates for such factors based on the previous studies mentioned above (17–28). The factors included sex (17, 18), BMI (17, 23), depression (19, 24, 25), anxiety (24), binge eating (18–20, 26), self-esteem (18, 19, 21, 27), and perfectionism (19, 22, 28).

## Materials and methods

### Study design

This retrospective analysis was done based on the physical and psychosocial data of 165 people with overweight (*n* = 73) or obesity (*n* = 92) who joined and completed our CBT-based weight loss treatment between 2012 and 2019 at the outpatient clinic for obesity of the Department of Psychosomatic Medicine at Kyushu University Hospital. Our treatment program consists of two phases, as previously described (29). The first phase is the weight loss phase. A CBT-based intensive weight loss program is provided in this phase, which lasts 7 months. Participants who lost at least 5% of their initial body weight during the weight loss phase entered the next phase we call the weight maintenance phase, which lasts 3 months. Of the 204 women who participated, 165 completed the 7-month weight loss phase. Of the 165 who entered the weight maintenance phase, 161 completed the weight maintenance phase. It should be noted that, for the present study, the data at baseline (before the weight loss phase) and at the end of the seventh month (at the end of the weight loss phase) were used. All patients had provided informed consent and their permission for the use of their anonymized data when signing up for the program. The Institutional Review Board of Kyushu University Hospital approved the study protocol.

### Eligibility

All patients were women, aged 20–65 years, with a BMI of 25 kg/m^2^ or higher. They were able to complete the self-report questionnaires without assistance. They had no physical impairment that would interfere with simple exercise. Exclusion criteria for the study were as follows: weight loss of > 5 kg during the previous 6 months, current diagnosis of BN, past history of anorexia nervosa, current pregnancy or breast feeding, planning to become pregnant within the next 24 months, taking any medication that would affect body weight, suffering from any health disorder affecting body weight, currently receiving treatment for psychiatric disorders or planning to move within the next 10 months. At their first visit, the principal investigator explained details of the CBT-based weight loss program to patients seeking weight loss treatment. Those who met the eligibility criteria were enrolled in the study. After informed consent was obtained, data on the patients’ demographics, lifestyle, and health history were collected.

### Weight loss intervention

Cooper et al. (9) developed the original CBT program for persons with obesity. We modified it for use in a group-therapy setting (30, 31). It includes the clinical guidelines on obesity of the US National Institutes of Health (32). Our program is conducted in groups of up to 10 people. It consists of 35 group sessions, each lasting 90 min, and five individual sessions over the 44-week period. Sessions are held once a week for the first 34 consecutive sessions, and once every 2 weeks for the rest of the sessions. The content of each session is shown in Supplementary Table 1. Two physicians and two certified nutritionists were involved in the treatment of the participants of this study. All participants were instructed to keep a daily food diary to keep track of their food and drink intake and to record the number of steps they took with a pedometer every day. They were also advised to consume a regular daily food diet, approximately 1,400–1,500 kcal on average, which was 500 kcal less than their baseline total calorie intake. The nutritionists examined their food diary and checked the nutritional balance of their diet. The participants were instructed to increase their exercise level to moderate intensity, such as walking (8000–10000 steps per day). A series of stress-management sessions was also included in the program, namely, lectures on cognitive reconstruction, problem solving, and assertion training. The last seven sessions of weight loss phase focused on BID, weight goals, and primary goals to prepare for the weight maintenance phase. In the intervention for BID, cognitive behavioral strategies were used to help those who tended to avoid body exposure, checked their body frequently, or had recurrent negative thoughts about their appearance (33). The goal of these strategies were: (i) To provide education about the role of body image in weight control and the development and maintenance of body image concerns, (ii) To identify body image concerns, (iii) To address these concerns, and (iv) To encourage the development of a positive body image (9). In every session, the participants were encouraged to engage in a cognitive-behavioral task related to that week’s theme and to record their attempts to perform the task on their monitoring records, together with associated thoughts. The outcome was then reviewed with an emphasis on whether or not the actual outcome was predicted by the participant (9). The main emphasis of the weight maintenance phase was on helping patients acquire the strategies and skills needed for long-term weight control, which included the regular monitoring of weight, the use of appropriate cognitive responses, and the practice of behavioral skills both to minimize significant weight fluctuations and to correct any significant changes which occur (9).

### Anthropometric measurements

Height and weight were measured and BMI was calculated at the beginning and at the end of the weight loss phase. %TWL (100 × weight loss/body weight) was calculated at the end of the weight loss phase.

### Psychosocial assessment

All participants completed a battery of self-reported psychosocial inventories before and at the end of the weight loss phase.

#### Body image dissatisfaction

The Body Shape Questionnaire (BSQ) was used to assess BID. The BSQ has demonstrated good reliability and validity in previous research (34, 35) and has been widely used in studies of BID for both non-clinical and clinical populations, including people with obesity. It is a 34-item self-report questionnaire, with each item scored from 1 (“never”) to 6 (“always”), giving a range from 34 to 204 for the sum of all items scores. Higher scores reflect greater BID. We used the Japanese version of BSQ, verified for reliability and validity by Kobayashi et al. (36).

#### Depression

The Japanese version of the Center for Epidemiologic Studies-Depression Scale (CES-D) was used to assess depression. The test–retest reliability and concurrent validity have been fully documented (37). CES-D is a 20-item self-report questionnaire. The score ranges from 0 to 60, with a higher score indicating the presence of more severe depressive symptoms (38).

#### Anxiety

The Japanese version of the State-Trait Anxiety Inventory (STAI) was used to assess anxiety (39). STAI is a self-reporting questionnaire consisting of two scales, with STAI-1 assessing state anxiety and STAI-2 assessing trait anxiety. Each scale consists of 20 items indicating the presence or absence of anxiety symptoms (40). The score of each scale ranges from 20 to 80, with higher score indicating higher anxiety.

#### Perfectionism

The Frost Multidimensional Perfectionism Scale (MPS-F) was used to evaluate perfectionism. MPS-F is a 35-item questionnaire that measures six dimensions of perfectionism (41): Concern over mistakes, Personal standards, Parental expectations, Parental criticism, and Doubts about actions, and Organization. Respondents rate their degree of agreement with various statements on a scale of 1 (strongly disagree) to 5 (strongly agree). A higher score indicates a higher level of perfectionism. The MPS-F has demonstrated adequate reliability (41) and validity, and the Japanese version has been validated (42).

#### Binge eating

The Binge Eating Scale (BES) was used to assess binge eating (43). It is a 16-item self-report questionnaire designed to measure the behavioral and emotional/cognitive symptoms associated with binge eating. Each item presents three or four differently weighted statements, with a final score varying from 0 to 46. The original BES has been translated into Japanese and back-translated by Sawamoto et al. (29). Although it has not yet been standardized, Cronbach’s alpha for the overall scale in the present study was 0.9419, which we considered to have good reliability.

### Statistical analysis

All statistical analyses were done with the JMP pro 15.1.0 software package (SAS Institute Inc., Cary, NC, USA). Changes in clinical data from before to after the weight loss phase were assessed by paired *t*-tests. Effect sizes were calculated using Cohen’s *d* and were classified as small (< 0.30), medium (0.30–0.80), and large (> 0.80) (44). The Pearson correlation coefficient test was used to examine the correlations of ΔBSQ with the change in body weight (percent total weight loss; %TWL), BSQ, CES-D, STAI-state, STAI-trait, BES, and MPS-F. We also assessed the correlations between ΔBSQ and the baseline data for these variables. A multiple regression analysis was performed using the forced entry method to examine factors that were independently associated with ΔBSQ. We conducted the multiple regression analysis with factors that were shown to be associated with ΔBSQ in the Pearson correlation coefficient test.

## Results

As shown in Table 1, significant decreases in body weight and BMI and improvement in the BSQ, BES, and MPS-F scales were noted at the end of the weight loss phase when compared to baseline, as shown by the large effect sizes (Cohen’s d) except for MPS-F. There were no significant changes in the scales of CES-D or the STAI. Pearson correlation coefficient test demonstrated that ΔBSQ was significantly associated with the following variables: baseline BSQ, baseline BES, %TWL, ΔCES-D, ΔSTAI-state, ΔSTAI-trait, ΔBES, and ΔMPS-F (Tables 2, 3). Multiple regression analysis showed that baseline BSQ, baseline BES, %TWL, ΔCES-D, and ΔBES were independently associated with ΔBSQ (Table 4).

**TABLE 1:** Changes in clinical data from before to after the weight loss phase.

Characteristics	Baseline			End of weight loss phase			*P*-value*	*d*
	**Mean**	**Max**	**Min**	**Mean (SD)**	**Max**	**Min**		

Age	49.3 ± 10.5	65.0	22.0					
Body weight (kg)	78.4 ± 14.0	139.9	56.0	67.2 ± 12.8	120.2	48.7	< 0.0001	0.84
BMI (kg/m^2^)	31.8 ± 5.2	53.5	25.1	27.3 ± 4.8	47.0	20.8	< 0.0001	0.90
% total weight loss				14.2 ± 5.3	33.0	4.0		0.90
BSQ	116.3 ± 30.2	195	45	96.9 ± 31.5	186	35	< 0.0001	0.63
CES-D	11.4 ± 7.7	44	0	11.5 ± 8.2	45	0	0.5519	0.01
STAI-state	42.0 ± 9.3	70	20	40.2 ± 10.3	75	20	0.082	0.18
STAI-trait	44.1 ± 11.7	72	20	43.8 ± 11.2	75	20	0.8193	0.03
BES	13.0 ± 6.8	39	0	7.0 ± 5.4	22	0	< 0.0001	0.98
MPS-F	88.3 ± 19.9	142	42	86.4 ± 18.3	159	45	0.0447	0.16

*Evaluated with the paired *t*-test. d: Cohen’s d.

Values are expressed as the means ± SD. BMI, body mass index. BSQ, Body Shape Questionnaire. CES-D, Center for Epidemiologic Studies-Depression Scale; STAI, State-Trait Anxiety Inventory; BES, Binge Eating Scale; MPS-F, total scale scores of Frost Multidimensional Perfectionism Scale.

**TABLE 2:** Correlations between ΔBSQ and baseline clinical variables.

	ΔBSQ	BMI	BSQ	CES-D	STAI-state	STAI-trait	BES	MPS-F
ΔBSQ	-							
BMI	0.0030	-						
BSQ	−0.3483***	0.2643***	-					
CES-D	–0.0577	0.1433	0.3673***	-				
STAI-state	–0.1220	0.1065	0.2831***	0.5651***	-			
STAI-trait	–0.0437	0.0694	0.3678***	0.6905***	0.6278***	-		
BES	−0.1881*	0.3247***	0.5062***	0.3126***	0.2112**	0.2897***	-	
MPS-F	–0.0158	0.1376	0.3523***	0.1899*	0.1290	0.3463***	0.2054**	-

*p < 0.05; **p < 0.01; ***p < 0.001.

Δ = values at the end of weight loss phase minus values at baseline. BSQ, Body Shape Questionnaire; BMI, Body mass index; CES-D, Center for Epidemiologic Studies-Depression Scale; STAI, State-Trait Anxiety Inventory; BES, Binge Eating Scale; MPS-F, total scale scores of Frost Multidimensional Perfectionism Scale.

**TABLE 3:** Correlations between ΔBSQ and change in clinical variables.

	ΔBSQ	%TWL	ΔCES-D	ΔSTAI-state	ΔSTAI-trait	ΔBES	ΔMPS-F
ΔBSQ	-						
%TWL	−0.2232**	-					
ΔCES-D	0.2710***	–0.0455	-				
ΔSTAI-state	0.2168**	–0.0118	0.4886***	-			
ΔSTAI-trait	0.1813*	–0.1232	0.4193***	0.5323***	-		
ΔBES	0.3999***	–0.1465	0.0923	0.1430	0.1857*	-	
ΔMPS-F	0.1777*	0.0758	0.2106**	0.1562	–0.0335	0.1189	-

*p < 0.05; **p < 0.01; ***p < 0.001.

Δ = values at the end of weight loss phase minus values at baseline. BSQ, Body Shape Questionnaire; %TWL, percent total weight loss; CES-D, Center for Epidemiologic Studies-Depression Scale; STAI, State-Trait Anxiety Inventory; BES, Binge Eating Scale; MPS-F, total scale scores of Frost Multidimensional Perfectionism Scale.

**TABLE 4:** Multiple regression analysis of ΔBSQ and clinical variables.

Independent variables	β	*p*
Age	0.0388	0.5815
Baseline BSQ	–0.4378	< 0.0001
Baseline BES	0.3401	0.0013
%TWL	–0.2051	0.0035
ΔCES-D	0.1772	0.0223
ΔSTAI-state	0.0692	0.3969
ΔSTAI-trait	–0.0634	0.4324
ΔBES	0.4910	< 0.0001
ΔMPS-F	0.0341	0.6233

β: standard regression coefficients.

Δ = values at the end of weight loss phase minus values at baseline.

*Age, baseline BSQ, baseline CES-D, baseline BES, %TWL, ΔCES-D, ΔSTAI-state, ΔSTAI-trait, ΔBES, and ΔMPS-F were entered as independent variables in a direct multiple regression model with ΔBSQ as the dependent variable. Adjusted *R*^2^ = 0.3687, *F* = 10.7353, *P* < 0.0001.

%TWL, percent total weight loss; BSQ, Body Shape Questionnaire; CES-D, Center for Epidemiologic Studies-Depression Scale; STAI, State-Trait Anxiety Inventory; BES, Binge Eating Scale; MPS-F, total scale scores of Frost Multidimensional Perfectionism Scale.

## Discussion and conclusion

This study examined if improvement of factors other than weight loss is associated with the improvement of BID during CBT for obesity. We found that not only weight loss, but also improvement of depression and binge eating were independently associated with improvement of BID. Thus, although no definitive conclusion can be made regarding causality, the results indicate that weight loss is not the only factor contributing to amelioration of BID during CBT for obesity. Consistent with the majority of previous studies (16, 45–49), improvement of BID was associated with weight loss. However, there have been a few other studies that found no association between weight loss and reduction in BID (50–52). One possible explanation for this inconsistency may be that these studies used different questionnaires to assess BID, such as the Multidimensional Body Self-Relations Questionnaire (53), Body Cathexis Scale (54), and Body Parts Satisfaction Scale (55). The use of these different questionnaires may have resulted in the assessment of different elements or different aspects of BID. However, the studies that used BSQ have consistently shown a positive correlation between weight loss and improvement of BID (23). Another explanation for the inconsistency is that there may be factors mediating the relation between weight loss and the improvement of BID. Ginis et al. reported that perceived changes to one’s body and perceived improvements in one’s physical abilities were more consistently correlated to BID change than were actual physique-related changes (56). Cernelic-Bizjak et al. showed a significant, yet weak, association between weight loss and change in BID, arguing that actual changes to the body may be less important than perceived changes (26). They also argued that other therapeutic or/and psychosocial aspects and the context of the intervention and/or personal life, in addition to weight loss, may have elicited body image improvements. In fact, this seems in line with a systematic review that revealed, though the studies were not limited to individuals with obesity, that intuitive eating, CBT, self-compassion, and exercise are helpful in promoting positive body image (57). Based on these, it seems reasonable to assume that how and to what extent one perceives the actual change to their body differ depending on the individual and the situation, leading to the discrepancies between the studies in terms of the relation between weight loss and improvement of BID. This study did not measure perceived change to the body, which will need to be addressed in future research.

The reduction of binge eating was associated with the amelioration of BID, even after controlling for weight change. Given that our CBT focuses on eating behavior before focusing on cognitive aspects such as BID and considering that, accordingly, participants’ eating behavior usually improves early in the intervention, it can be assumed that improvement of binge eating influenced the reduction of BID. Again, it is hypothesized that there is a mediating factor between reduction of BID and improvement of binge eating. As we have discussed, BID may be affected if there is a change in the way one perceives the actual change to the body, and it is likely that such a change depends on the context of weight loss, such as the clinical course of the binge eating. In other words, the fact that a patient successfully improved binge eating might have had a positive impact on the way she looks at her body, leading to the amelioration of BID. This hypothesis seems to be in line with previous studies that examined factors associated with BID. In a cross-sectional study that assessed bariatric surgery candidates with extreme obesity, Bianciardi et al. demonstrated that the “need for approval” dimension of insecure attachment style was an independent predictor of BID (58). It seems theoretically possible that a certain dimension of insecure attachment style affects the way one looks at one’s body, thereby influencing BID. In a longitudinal study, Annesi et al. found that treatment type and increased physical activity was associated with improved body satisfaction, even after controlling for weight changes (59); It is possible that treatment settings (e.g., group vs. individual) and physical activity have an impact on the way one looks at one’s body, which would affect change in BID. In this light, factor(s) that either represent or influence the way one looks at one’s body seem to be a worthwhile direction of research inquiry. One such possible factor is self-esteem. This is because, although in cross-sectional studies for bariatric surgery candidates, self-esteem has been shown to be correlated to both BID (18, 19) and binge eating (60, 61). Also, theoretically, it may function as a measure of the way one looks at one’s body. Another possible factor is self-efficacy; it has been shown that changes of self-efficacy predicted variance in change of BID (56), and it can also theoretically influence the way one looks at one’s body. Unfortunately, the present study did not examine either of these factors. Future research will be necessary to examine the possible mediating roles of self-esteem and self-efficacy, in longitudinal studies of people with obesity who are not candidates for bariatric surgery.

Another important consideration related to binge eating is that the mean BES score at baseline of 13.0 ± 6.8 was below 18, the cut-off score suggestive of BED. In fact, we had 36 participants (21.8%) whose baseline BES scores were 18 or higher (data not shown). Regarding this, we found that participants with baseline BES scores of 18 or higher had score reductions on a wide range of items, while for those with baseline BES scores below 18 the score reductions tended to be seen on items related to loss-of-control eating (LOC eating; the subjective sense that one cannot stop eating or control what or how much one is eating) (62–64) (Supplementary Tables 2, 3). For those who had a baseline BES score below 18 and were unlikely to meet the criteria for BED, CBT was useful in reducing their LOC eating.

The improvement of depression was associated with the amelioration of BID. Although we did not examine this, it is possible that improvement of self-esteem mediated the relation between improvement in depression and BID; previous studies have reported a positive association between improvement in depression and BID in people who underwent bariatric surgery (65, 66). It should be mentioned that depression scores showed no significant changes after treatment. Additional analyses showed that, in the subgroup of participants who had a median or higher level of depression scores at baseline, the improvement in BID was significantly greater in those with an improvement in depression scores than those without (data not shown).

Regarding perfectionism, previous studies assessed either the cross-sectional relation between perfectionism and BID (19, 22) or the predictive function of perfectionism at baseline for the post-CBT score of BID (28), as we have mentioned. However, the mechanism of the relation between BID and perfectionism has not been fully discussed. It may be that people with high perfectionism have high standards of body image, which can highlight the discrepancy between the ideal body and the actual body, leading to their high BID scores. The present study assessed the relation between the improvement of perfectionism and the amelioration of BID and found that, although these two factors are correlated, they are not independently associated when weight loss, baseline BID, depression, anxiety, binge eating, and perfectionism data, and improvements of these variables are controlled for. Thus, this implies that improvement of perfectionism does not play a central role in the amelioration of BID.

A strength of our study is the longitudinal nature of our study design. To the best of our knowledge, this is the first longitudinal study that assesses associations between improvement of BID and changes in other psychological factors such as depression, anxiety, and perfectionism, and eating-related factors such as binge eating during weight loss treatment. Our study includes several limitations. First, because of the study design, the results cannot be used to determine a causal relation between BID and psychological factors such as binge eating or depression. Second, we did not include self-esteem and self-efficacy as possible correlates of BID, despite their previously shown associations with BID. Third, the participants were all Japanese women, thus the findings cannot be generalized to patients with obesity in general.

In conclusion, the present study indicated that improvement of binge eating and depression may bring about a reduction of BID. Focusing on amelioration of binge eating and depression as well as weight loss during CBT for obesity may improve BID and contribute to long-term weight maintenance.

## Data availability statement

The raw data supporting the conclusions of this article will be made available by the authors, without undue reservation.

## Ethics statement

The studies involving human participants were reviewed and approved by the Institutional Review Board of Kyushu University Hospital. The patients/participants provided their written informed consent to participate in this study.

## Author contributions

HY and TaN contributed to the conception of the work. HY conducted the data analyses and prepared the manuscript. ToN, RS, and TaN participated in the weight loss and maintenance intervention. TaN assisted with the data analysis and preparation of the manuscript. GK and NS made substantial contribution to the final manuscript. All authors interpreted the findings and contributed to the writing, editing, and approval of the final draft.

## References

[1] Leite PB Dâmaso AR Poli VS Sanches RB Silva SGA Fidalgo JPN Long-term interdisciplinary therapy decreases symptoms of binge eating disorder and prevalence of metabolic syndrome in adults with obesity. *Nutr Res.* (2017) 40:57–64. 10.1016/j.nutres.2017.03.006 28473061

[2] Grave RD Sartirana M Ghoch MEl Calugi S. *Treating Obesity with Personalized Cognitive Behavioral Therapy.* Cham: Springer International Publishing (2018). p. 1–244.

[3] Mitchison D Hay P Griffiths S Murray SB Bentley C Gratwick-Sarll K Disentangling body image: the relative associations of overvaluation, dissatisfaction, and preoccupation with psychological distress and eating disorder behaviors in male and female adolescents. *Int J Eat Disord.* (2017) 50:118–26. 10.1002/eat.22592 27539911 PMC6585604

[4] Hagan KE Walsh BT. State of the art: the therapeutic approaches to bulimia nervosa. *Clin Ther.* (2021) 43:40–9. 10.1016/j.clinthera.2020.10.012 33358256 PMC7902447

[5] Grilo CM. Psychological and behavioral treatments for binge-eating disorder. *J Clin Psychiatry.* (2017) 78(Suppl. 1):20–4. 10.4088/JCP.sh16003su1c.04 28125175

[6] Anderson DA Maloney KC. The efficacy of cognitive-behavioral therapy on the core symptoms of bulimia nervosa. *Clin Psychol Rev.* (2001) 21:971–88. 10.1016/S0272-7358(00)00076-3 11584518

[7] Ghaderi A Odeberg J Gustafsson S Rastam M Brolund A Pettersson A Psychological, pharmacological, and combined treatments for binge eating disorder: a systematic review and metaanalysis. *PeerJ.* (2018) 6:e5113. 10.7717/peerj.5113 29942715 PMC6015752

[8] Hilbert A Tuschen-Caffier B. Body image interventions in cognitive-behavioural therapy of binge-eating disorder: a component analysis. *Behav Res Ther.* (2004) 42:1325–39. 10.1016/j.brat.2003.09.001 15381441

[9] Cooper Z Fairburn CG Hawker DM. *Cognitive–Behavioral Treatment of Obesity; a Clinician’s Guide.* New York, NY: Guilford Press (2003).

[10] Cooper Z Doll HA Hawker DM Byrne S Bonner G Eeley E Testing a new cognitive behavioural treatment for obesity: a randomized controlled trial with three-year follow-up. *Behav Res Ther.* (2010) 48:706–13. 10.1016/j.brat.2010.03.008 20691328 PMC2923743

[11] Jacob A Moullec G Lavoie KL Laurin C Cowan T Tisshaw C Impact of cognitive-behavioral interventions on weight loss and psychological outcomes: a meta-analysis. *Heal Psychol.* (2018) 37:417–32. 10.1037/hea0000576 29698017

[12] Ashton K Drerup M Windover A Heinberg L. Brief, four-session group CBT reduces binge eating behaviors among bariatric surgery candidates. *Surg Obes Relat Dis.* (2009) 5:257–62. 10.1016/j.soard.2009.01.005 19250884

[13] Chao HL. Body image change in obese and overweight persons enrolled in weight loss intervention programs: a systematic review and meta-analysis. *PLoS One.* (2015) 10:e0124036. 10.1371/journal.pone.0124036 25946138 PMC4422747

[14] Dalle Grave R Sartirana M Calugi S. Personalized cognitive-behavioural therapy for obesity (CBT-OB): theory, strategies and procedures. *Biopsychosoc Med.* (2020) 14:1–9. 10.1186/s13030-020-00177-9 32175002 PMC7063798

[15] Santos I Mata J Silva MN Sardinha LB Teixeira PJ. Predicting long-term weight loss maintenance in previously overweight women: a signal detection approach. *Obesity.* (2015) 23:957–64. 10.1002/oby.21082 25865433

[16] Teixeira PJ Silva MN Coutinho SR Palmeira AL Mata J Vieira PN Mediators of weight loss and weight loss maintenance in middle-aged women. *Obesity.* (2010) 18:725–35. 10.1038/oby.2009.281 19696752

[17] Weinberger NA Kersting A Riedel-Heller SG Luck-Sikorski C. Body dissatisfaction in individuals with obesity compared to normal-weight individuals: a systematic review and meta-analysis. *Obes Facts.* (2017) 9:424–41. 10.1159/000454837 28013298 PMC5644896

[18] Grilo CM Masheb RM Brody M Burke-Martindale CH Rothschild BS. Binge eating and self-esteem predict body image dissatisfaction among obese men and women seeking bariatric surgery. *Int J Eat Disord.* (2005) 37:347–51. 10.1002/eat.20130 15856499

[19] Rosenberger PH Henderson KE Grilo CM. Correlates of body image dissatisfaction in extremely obese female bariatric surgery candidates. *Obes Surg.* (2006) 16:1331–6. 10.1381/096089206778663788 17059743

[20] De Zwaan M Mitchell JE Howell LM Monson N Swan-Kremeier L Crosby RD Characteristics of morbidly obese patients before gastric bypass surgery. *Compr Psychiatry.* (2003) 44:428–34. 10.1016/S0010-440X(03)00092-0 14505305

[21] Matz PE Foster GD Faith MS Wadden TA. Correlates of body image dissatisfaction among overweight women seeking weight loss. *J Consult Clin Psychol.* (2002) 70:1040–4. 10.1037/0022-006X.70.4.1040 12182267

[22] Dunkley DM Blankstein KR Masheb RM Grilo CM. Personal standards and evaluative concerns dimensions of “clinical” perfectionism: a reply to Shafran etal. (2002, 2003) and Hewitt etal. (2003). *Behav Res Ther.* (2006) 44:63–84. 10.1016/j.brat.2004.12.004 16301015

[23] Lasikiewicz N Myrissa K Hoyland A Lawton CL. Psychological benefits of weight loss following behavioural and/or dietary weight loss interventions. A systematic research review. *Appetite.* (2014) 72:123–37. 10.1016/j.appet.2013.09.017 24075862

[24] Geller S Dahan S Levy S Goldzweig G Hamdan S Abu-Abeid S. Body image and emotional eating as predictors of psychological distress following bariatric surgery. *Obes Surg.* (2020) 30:1417–23. 10.1007/s11695-019-04309-1 31823307

[25] Behrens SC Lenhard K Junne F Ziser K Lange J Zipfel S Effects of bariatric surgery on depression: role of body image. *Obes Surg.* (2021) 31:1864–8. 10.1007/s11695-020-05057-3 33089383 PMC8012322

[26] Černelič-Bizjak M. Changes in body image during a 6-month lifestyle behaviour intervention in a sample of overweight and obese individuals. *J Bodyw Mov Ther.* (2019) 23:515–20. 10.1016/j.jbmt.2019.01.015 31563364

[27] Crerand CE Wadden TA Foster GD Sarwer DB Paster LM Berkowitz RI. Changes in obesity-related attitudes in women seeking weight reduction. *Obesity.* (2007) 15:740–7. 10.1038/oby.2007.590 17372325

[28] Malkina-Pykh IG. Effectiveness of rhythmic movement therapy for disordered eating behaviors and obesity. *Span J Psychol.* (2012) 15:1371–87. 10.5209/rev_SJOP.2012.v15.n3.39422 23156940

[29] Sawamoto R Nozaki T Nishihara T Furukawa T Hata T Komaki G Predictors of successful long-term weight loss maintenance: a two-year follow-up. *Biopsychosoc Med.* (2017) 11:14. 10.1186/s13030-017-0099-3 28592990 PMC5460352

[30] Nozaki T Sawamoto R Sudo N. Cognitive behavioral therapy for obesity. *Nihon Rinsho.* (2013) 71:329–34. 23631216

[31] Nozaki T Sawamoto R Furuwaka T Tanahashi T Morita C Hata T Group based cognitive behavioral therapy for obesity. *Jpn J Psychosm Int Med.* (2013) 17:220–5.

[32] North American Association for the Study of Obesity. Executive summary. *Obes Res.* (1998) 6(Suppl. 2):51S–179S. 10.1002/j.1550-8528.1998.tb00690.x9813653

[33] Cooper Z Fairburn CG. A new cognitive behavioural approach to the treatment of obesity. *Behav Res Ther.* (2001) 39:499–511. 10.1016/S0005-7967(00)00065-6 11341247

[34] Cohen E Ndao A Bernard JY Gueye A Duboz P Macia E Development and validation of the body shape scale (BOSHAS) for assessing body shape perception in African populations. *BMC Public Health.* (2020) 20:1562. 10.1186/s12889-020-09654-w 33066748 PMC7566052

[35] Rosen JC Jones A Ramirez E Waxman S. Body shape questionnaire: studies of validity and reliability. *Int J Eat Disord.* (1996) 20:315–9. 10.1002/(SICI)1098-108X(199611)20:3<315::AID-EAT11>3.0.CO;2-Z 8912044

[36] Kobayashi Y Tachi T Murotsu K Fukuchi Y. Clinical trial of body shape questionnaire(BSQ) in eating disorder patients; reliability and validity of the Japanese version of BSQ. *Jpn J Clin Psychiatry.* (2001) 30:1501–8.

[37] Shima S Shikano T Kitamura T Asai M. A new self-report depression scale. *Clin Psychiatry.* (1985) 27:1985.

[38] Radloff LS. The CES-D scale: A self-report depression scale for research in the general population. *Appl Psychol Meas.* (1977) 1:385–401. 10.1177/014662167700100306 26918431

[39] Nakazato K Mizuguchi K. Studies on psychometric characteristics of depression in the field of internal medicine. *Jap J Psychosom Med.* (1982) 22:107–12.

[40] Spielberger C Gorsuch R Lushene R Vagg P Jacobs G. *Manual for the State- Trait Anxiety Inventory (STAI).* Palo Alto, CA: Consult Psychol Press (1983).

[41] Frost RO Marten P Lahart C Rosenblate R. The dimensions of perfectionism. *Cogn Ther Res.* (1990) 14:449–68. 10.1007/BF01172967

[42] Tanaka H Nagata T Kiriike N Kawarada Y Matsunaga H Yamagami S. Perfectionism in patients with eating disorders. *Seishin Igaku (Clin Psychiatry).* (1999) 41:847–53. 10.11477/mf.1405904821

[43] Gormally J Black S Daston S Rardin D. The assessment of binge eating severity among obese persons. *Addict Behav.* (1982) 7:47–55. 10.1016/0306-4603(82)90024-7 7080884

[44] Cohen J. *Applied Multiple Regression/Correlation Analysis for the Behavioral Sciences.* New York, NY: Routledge (2003).

[45] Palmeira AL Markland DA Silva MN Branco TL Martins SC Minderico CS Reciprocal effects among changes in weight, body image, and other psychological factors during behavioral obesity treatment: a mediation analysis. *Int J Behav Nutr Phys Act.* (2009) 6:1–12. 10.1186/1479-5868-6-9 19203389 PMC2645358

[46] Palmeira AL Branco TL Martins SC Minderico CS Silva MN Vieira PN Change in body image and psychological well-being during behavioral obesity treatment: associations with weight loss and maintenance. *Body Image.* (2010) 7:187–93. 10.1016/j.bodyim.2010.03.002 20409769

[47] Teixeira PJ Going SB Houtkooper LB Cussler EC Metcalfe LL Blew RM Exercise motivation, eating, and body image variables as perdictors of weight control. *Med Sci Sports Exerc.* (2006) 38:179–88. 10.1249/01.mss.0000180906.10445.8d 16394972

[48] Kiernan M King AC Stefanick ML Killen JD. Men gain additional psychological benefits by adding exercise to a weight-loss program. *Obes Res.* (2001) 9:770–7. 10.1038/oby.2001.106 11743061

[49] Messier V Rabasa-Lhoret R Doucet E Brochu M Lavoie J-M Karelis A Effects of the addition of a resistance training programme to a caloric restriction weight loss intervention on psychosocial factors in overweight and obese post-menopausal women: a Montreal Ottawa new emerging team study. *J Sports Sci.* (2010) 28:83–92. 10.1080/02640410903390105 20035493

[50] Foster GD Wadden TA Vogt RA. Body image in obese women before, during, and after weight loss treatment. *Heal Psychol.* (1997) 16:226–9. 10.1037/0278-6133.16.3.226 9152700

[51] Rippe JM Price JM Hess SA Kline G DeMers KA Damitz S Improved psychological well-being, quality of life, and health practices in moderately overweight women participating in a 12-week structured weight loss program. *Obes Res.* (1998) 6:208–18. 10.1002/j.1550-8528.1998.tb00339.x 9618125

[52] Bas M Donmez S. Self-efficacy and restrained eating in relation to weight loss among overweight men and women in Turkey. *Appetite.* (2009) 52:209–16. 10.1016/j.appet.2008.09.017 18929608

[53] Cash TF. *The MBSRQ Users’ Manual.* 3rd ed. Norfolk, VA: Old Dominion University Press (2000).

[54] Slade PD Dewey ME Newton T Brodie D Kiemle G. Development and preliminary validation of the body satisfaction scale (BSS). *Psychol Health.* (1990) 4:213–20. 10.1080/08870449008400391

[55] Petrie TA Tripp MM Harvey P. Factorial and construct validity of the body parts satisfaction scale-revised: an examination of minority and nonminority women. *Psychol Women Q.* (2002) 26:213–21. 10.1111/1471-6402.00060

[56] Martin Ginis KA McEwan D Josse AR Phillips SM. Body image change in obese and overweight women enrolled in a weight-loss intervention: the importance of perceived versus actual physical changes. *Body Image.* (2012) 9:311–7. 10.1016/j.bodyim.2012.04.002 22608984

[57] Guest E Costa B Williamson H Meyrick J Halliwell E Harcourt D. The effectiveness of interventions aiming to promote positive body image in adults: a systematic review. *Body Image.* (2019) 30:10–25. 10.1016/j.bodyim.2019.04.002 31077956

[58] Bianciardi E Di Lorenzo G Niolu C Betrò S Zerbin F Gentileschi P Body image dissatisfaction in individuals with obesity seeking bariatric surgery: exploring the burden of new mediating factors. *Riv Psichiatr.* (2019) 54:8–17. 10.1708/3104.30935 30760932

[59] Annesi JJ. Mediation of the relationship of behavioural treatment type and changes in psychological predictors of healthy eating by body satisfaction changes in women with obesity. *Obes Res Clin Pract.* (2017) 11:97–107. 10.1016/j.orcp.2016.03.011 27085768

[60] Cella S Fei L D’Amico R Giardiello C Allaria A Cotrufo P. Binge eating disorder and related features in bariatric surgery candidates. *Open Med.* (2019) 14:407–15. 10.1515/med-2019-0043 31231682 PMC6572385

[61] Özdin S Karabekiroğlu A Özbalcı GS Aker AA. The effect of cognitive symptoms in binge eating disorder on depression and self-esteem: a cross-sectional study. *Eat Weight Disord.* (2020) 26:1483–9. 10.1007/s40519-020-00966-9 32691335

[62] Ivezaj V Kessler EE Lydecker JA Barnes RD White MA Grilo CM. Loss-of-control eating following sleeve gastrectomy surgery. *Surg Obes Relat Dis.* (2017) 13:392–8. 10.1016/j.soard.2016.09.028 27913121 PMC5357454

[63] Grupski AE Hood MM Hall BJ Azarbad L Fitzpatrick SL Corsica JA. Examining the binge eating scale in screening for binge eating disorder in bariatric surgery candidates. *Obes Surg.* (2013) 23:1–6. 10.1007/s11695-011-0537-4 23104387 PMC4874644

[64] Hood MM Grupski AE Hall BJ Ivan I Corsica J. Factor structure and predictive utility of the binge eating scale in bariatric surgery candidates. *Surg Obes Relat Dis.* (2013) 9:942–8. 10.1016/j.soard.2012.06.013 22963818 PMC4874331

[65] Preiss K Clarke D O’Brien P de la Piedad Garcia X Hindle A Brennan L. Psychosocial predictors of change in depressive symptoms following gastric banding surgery. *Obes Surg.* (2018) 28:1578–86. 10.1007/s11695-017-3055-1 29423556

[66] Felske AN Williamson TM Scurrey SRM Telfer JA Campbell TS Rash JA. The influence of weight-related self-esteem and symptoms of depression on shape and weight concerns and weight-loss 12 months after bariatric surgery. *Obes Surg.* (2021) 31:1062–72. 10.1007/s11695-020-05097-9 33185838

